# Spot urine iodine levels below the WHO recommendation are not related to impaired thyroid function in healthy children and adolescents

**DOI:** 10.1007/s00394-020-02268-3

**Published:** 2020-05-11

**Authors:** Tillmann Wallborn, Mandy Vogel, Antje Kneuer, Michael Thamm, Katalin Dittrich, Wieland Kiess, Jürgen Kratzsch

**Affiliations:** 1 University Hospital for Children and Adolescents, University of Leipzig, 04103 Leipzig, Germany; 2 LIFE Research Center for Civilization Diseases, University of Leipzig, Leipzig, Germany; 3 Department of Epidemiology and Health Monitoring, Robert Koch Institute, 13302 Berlin, Germany; 4 Institute of Laboratory Medicine, Clinical Chemistry and Molecular Diagnostics, University Hospital, Leipzig, Germany

**Keywords:** Iodine, Thyroid, Urine, Children, TSH, FT4, FT3

## Abstract

**Purpose:**

Iodine deficiency in childhood and adolescence may lead to later thyroid dysfunction, stunted growth and cognitive impairment. The World Health Organization (WHO) has issued recommended age-dependent urine iodine concentration targets, but a critical threshold beyond which clinical sequelae are to be expected remains undefined. Our study aimed to investigate spot urine iodine concentration in a typical Central European cohort of children and adolescents, and consider the implications of these values in regard to laboratory parameters for evaluating thyroid function.

**Methods:**

Using the Sandell-Kolthoff method, spot urine iodine concentration was measured cross-sectionally from 1802 healthy children and adolescent in the age range of 0.25–18 years within the LIFE-Child epidemiological study based in and around the city of Leipzig (Germany). Additionally, serum thyroid biomarkers of these subjects were measured and correlated to urine iodine levels.

**Results:**

In our cohort, 61.39% of boys and 65.91% of girls had an iodine level of < 100 µg/L (57%, 67%, 65% of the age groups 0–5, 6–12 and 13–18 years), the median iodine excretion was 86 µg/L in boys and 80 µg/L in girls. The iodine levels revealed no significant correlation with the thyroid biomarkers TSH, FT4 and FT3. Moreover, iodine values revealed no correlation with levels of antibodies against thyroid peroxidase or thyroglobulin.

**Conclusion:**

In our cohort of children and adolescents, the relatively high number of iodine levels below the WHO recommendation appears not to be related to clinical or subclinical thyroid diseases in the respective participants.

## Introduction

The World Health Organization (WHO) recommended daily intake of iodine for children and adolescents of 90–150 µg/day, depending on age [1, 2]. Recommendations given from German authorities were in a comparable range of 40–200 µg/day [3]. Numerous clinical studies suggested that insufficient iodine intake can lead to hypothyroidism with thyroid enlargement (goiter), stunted growth, cognitive impairment (especially of the fetal brain), an increased risk of stillbirth, or a number of other typical clinical signs [4]. Accordingly, iodine deficiency may be considered one of the most significant single causes of preventable brain damage in the world [5].

In recent years, iodine fortification of foodstuffs has been used worldwide as a key strategy in addressing the problem of iodine deficiency. By 2011, the number of iodine-deficient countries had decreased from 54 to 32 as compared to the year 2003, while the number of countries with adequate supply increased from 67 to 105 [6]. Despite this positive development, it has been hypothesized that 29% of the world’s population has an insufficient iodine intake [7]. This problem is not only a question of socioeconomic development. Even in a number of high-income countries (e.g. in Europe) the risk of iodine deficiency is higher than in some poorer regions of the world [8–10]. The WHO reported an improved iodine supply in European children, based on a decrease in levels of iodine deficiency from 60% of children in 2003 to 44% in 2012 [4, 7, 11]. In 2005, the WHO suggested that the state of iodine nutrition be reported every three years (WHA58.24), but global data about progress in resolving iodine deficiency are incomplete. The most recent report about the prevalence of iodine deficiency in Germany was only published in 2006 [11]. A nationwide study showed 41% of children and adolescents with suboptimal iodine intake in the period from 2003 to 2006 with the highest portion of both, insufficient and excessive iodine intake in the age group 0–2 years [12]. This is a significantly higher percentage than the 27% reported by a former survey in Germany from 1999 [13]. The observation of increased levels of iodine deficiency agrees with another German nationwide food habit survey (“Nationale Verzehrstudie II”) carried out in 2005 and 2006. This study revealed that a high percentage of adolescents—41.1% of adolescent boys and 73.1% of adolescent girls—had a daily iodine intake of less than 200 µg [14].

To date, a number of different methods have been described for diagnosing iodine deficiency. In the past, thyroid function impairment was investigated by palpation of the thyroid gland or by the detection of increased thyroid volume via ultrasound. However, these methods were limited in terms of both sensitivity and specificity, especially in cases of subclinical diseases [15]. Moreover, both methods were susceptible to severe inter-observer error. Thus, the WHO recommended the measurement of iodine concentration in spot urine samples (SUIC) as an objective method for estimating iodine intake [2]. SUIC appears to be an effective indicator of recent dietary iodine intake since > 92% of ingested iodine is absorbed and > 90% is excreted within 24–48 h [15]. Since iodine excretion generally demonstrated a large intra-day variance, more accurate results can be obtained by collecting urine over a 24-h period. However, this method was not practicable in large field studies, especially those involving infants and children [15]. Surveys of iodine status have usually been carried out with cohorts of school children because they are easily accessible and representative for the general population [13]. The WHO provided the following reference concentration ranges for median SUIC: severe iodine deficiency < 20 µg/L, moderate iodine deficiency 20–49 µg/L, mild iodine deficiency 50–99 µg/L, adequate iodine nutrition 100–199 µg/L, above requirements 200–299 µg/L, excessive (posing risk of adverse health consequences) ≥ 300 µg/L [2]. According to the WHO, in a population not characterized by iodine deficiency, the *median* SUIC should be between 100–299 μg/l in children, with no more than 20% of children exhibiting an SUIC below 50 μg/L. The median was used instead of the mean because the distribution of SUIC levels is often skewed towards higher levels [16].

Although the pathophysiological relationship between iodine deficiency and thyroid dysfunction has been clearly established, no critical threshold has been derived from a population-based SUIC sample below which we can say there is a high probability of developing thyroid dysfunction. Moreover, various studies have indicated that an excessive intake of iodine may increase the incidence of autoimmune thyroiditis [17]. High levels of iodine may modulate the immune system and cause the destruction of thyrocytes. In consequence, higher rates of hypothyroidism can be found in regions with high iodine intake. An association between high SUIC (> 300 g/L) and autoimmune thyroiditis (thyroid peroxidase antibodies) has also been found in children [18]. The aim of our study was, first, to investigate SUIC levels among children and adolescents in the LIFE Child study based in Leipzig (Germany) and, second, to consider whether these SUIC values might show any correlation with parameters of thyroid function in our cohort.

## Subjects

Our cohort of infants, children and adolescents was recruited from among the participants of the LIFE Child study in Leipzig (clinical trial number NCT02550236). This study is a prospective, longitudinal health survey initiated by the Hospital for Children and Adolescents at the University of Leipzig (Germany). The study is open to all children and adolescents (and their parents) and includes medical and psychological examinations and questionnaires. Age of study participants was between 3 months and 17 years of age (median 9.4 years). In terms of their anthropometric data, our cohort was representative of the general population (mean height-SDS 0.12, mean BMI-SDS 0.13). Participants are requested to come without having eaten breakfast to the study clinic. After a medical examination, blood and spot urine samples were withdrawn via urine cup, urine potty or urine bag. A detailed description of the LIFE Child study, including the recruitment process and data acquisition, is available [19, 20]. The study was approved by the Ethics Committee of the University of Leipzig (Reg. No. 264-10-19042010). Fully informed, written consent was obtained from all participants and their parents after a full explanation of the purpose and nature of all procedures used. All of the samples and data on which our study is based were collected between 11/2013 and 08/2015 in a cross-sectional setting. One blood as well as one urine sample of the first visit of each participant was included in the study archive. An aliquot of the total urine sample of each study subject was stored at − 80 °C in a local biobank. Samples were sent to the analyzing laboratory (Robert Koch Institute, Central Epidemiological Laboratory, Berlin) and were stored at − 40 °C before analysis.

In total, we included 1982 subjects with completed questionnaires and available spot urine samples. Children and adolescents with endocrine, metabolic, renal, cardiac, gastrointestinal or muscular diseases were excluded. Moreover, subjects who were taking medication (e.g. thyroid hormones or antibiotics) or whose medical examination produced abnormal findings (fever, goiter) were also excluded. The remaining sample comprised of 1802 individual subjects.

## Methods

Inductively coupled plasma mass spectrometry (ICP-MS) is the current gold standard for iodine measurement but this method is prohibitively complex and costly [21]. Therefore, in line with the WHO recommendation, SUIC values were measured using the Sandell-Kolthoff method [2]. To remove interfering substances, a 500 µL sample was mixed with 800 µL ammonium peroxydisulfate (1 mol/L) and 200 µL sodium hydroxide (0.875 mol/L) and heated for 60 min to 95 ± 2 °C (thermoblock, Rotilabo^®^ Block-Heater H250, Roth, Karlsruhe, Germany) [22]. Thereafter, samples were cooled to room temperature and transferred to the analyzer (Cobas Mira plus, Roche, Basel, Switzerland). An aliquot of 80 µL of digested sample was mixed with 200 µL arsenious acid (0.0253 mol/L) and after 168 s, the Sandell-Kolthoff reaction was initiated using 50 µL Cerium(IV)-sulfate (0.0378 mol/L). The detection range of the method included SUIC values from 7.5 µg/L up to 419 µg/L. The method was cross-validated with ICP-MS [21]. All other laboratory parameters were determined using a Cobas automated analyzer (Roche, Mannheim): creatinine was measured by enzymatic method, TSH (thyrotropin), FT3 (free triiodthyronine), FT4 (free thyroxine), TPO-Ab (thyroid peroxidase antibodies) and TG-Ab (thyroglobulin antibodies) were determined by the electrochemiluminescence method (ECLIA). Reference ranges for TSH, FT3 and FT4 were established by an in-house study [23]. To investigate correlations between thyroid biomarkers, creatinine and iodine concentrations, age and gender-adjusted standard deviation scores (SDS) were determined for TSH, FT3 and FT4.

Creatinine is an indicator of the individual’s hydration status. For creatinine adjustment of SUIC the ratio of urinary iodine to creatinine (creatinine-corrected iodine, CCUI) was calculated. CCUI values were converted to age- and sex-adjusted SDS.

Descriptive statistics were determined in terms of median and interquartile range for iodine, TPO-Ab and TG-Ab as well as of mean and standard deviation for TSH, FT3 and FT4. Univariate relationships according to Pearson were estimated between iodine status and thyroid function. Empirical density was estimated using a Gaussian kernel. Group differences were tested applying 2-sample *t* tests. Measurement values were converted to standard deviation scores (SDS) using the extended LMS-method as implemented in the gamlss package [24]. All statistical analysis and data visualization were performed using the statistical language R [25]. The significance level was set to alpha = 0.05.

## Results

### Spot urine iodine levels

**Fig. 1:**
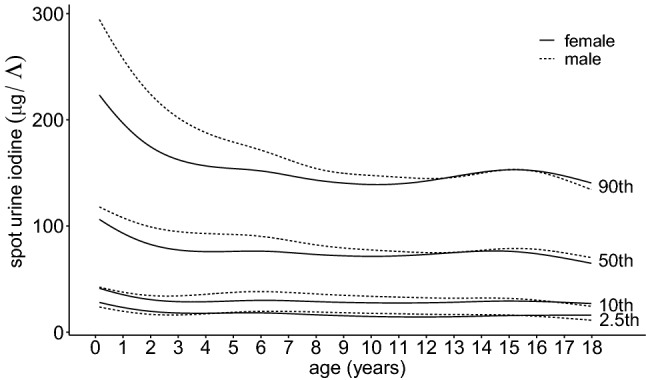
Spot urine iodine concentration of all participants by age and gender with percentiles

The median SUIC in our cohort was 86.3 µg/L for boys (*n* = 1010) and 80.0 µg/L for girls (*n* = 792) without a significant difference between gender (Table 1; Fig. 1). The highest levels of iodine were measured in the first year of life with a non-significantly higher median SUIC of 112.3 µg/L in boys compared to 106.0 µg/L in girls. Thereafter, SUIC values present a trend to lower levels, especially in boys. The correlation between age and iodine was not significant in our correlation model, but significant in the linear regression model. Also interquartile ranges were higher in the first year of age with a negative SUIC trend during childhood, again especially in boys. In our cohort in total 61.39% of boys and 65.91% of girls presented iodine values below 100 µg/L, the threshold for iodine deficiency according to the WHO classification (Table 2). Percentage of subjects with severe iodine deficiency (< 20 µg/L) was 4.06% in boys and 4.42% in girls. Only 13 participants (0.72%) showed iodine levels in the “excessive” range of > 300 µg/L.

**Table 1:** Study cohort: overview of age and gender distribution and results of laboratory analysis

Age group	Male	Female	Urine iodine (μg/L)		Urine creatinine (μmol/L)		TSH (mU/L)		FT4 (pmol/L)		FT3 (pmol/L)	
			Male	Female	Male	Female	Male	Female	Male	Female	Male	Female
(years)	*N*	*N*	Median (IQR)	Median (IQR)	Mean ± SD	Mean ± SD	Mean ± SD	Mean ± SD	Mean ± SD	Mean ± SD	Mean ± SD	Mean ± SD
0	122	44	112.3 (128.2)	106.0 (69.5)	1620 ± 1211	1106 ± 710	3.7 ± 1.5	3.6 ± 1.5	16.6 ± 1.8	16.8 ± 1.5	7.0 ± 0.6	7.3 ± 0.8
1	24	19	94.5 (112.3)	67.3 (55.2)	3480 ± 3735	1967 ± 1056	2.7 ± 0.7	2.7 ± 0.7	16.6 ± 1.4	16.4 ± 1.3	7.0 ± 0.8	7.0 ± 0.8
2	36	30	86.3 (77.1)	73.6 (63.1)	3882 ± 2897	3434 ± 2369	2.6 ± 1.0	3.0 ± 1.1	16.1 ± 1.6	16.0 ± 1.4	6.9 ± 0.9	6.7 ± 0.7
3	45	40	110.4 (93.9)	76.1 (80.6)	4990 ± 2646	4710 ± 2694	2.8 ± 0.9	2.7 ± 1.1	16.2 ± 1.6	16.1 ± 1.6	6.6 ± 0.7	7.1 ± 1.2
4	51	37	88.8 (71.1)	72.3 (50.8)	7138 ± 3602	5278 ± 2874	2.8 ± 10.5	2.6 ± 1.2	16.4 ± 1.4	16.5 ± 1.8	6.8 ± 0.7	6.7 ± 0.6
5	56	53	97.1 (45.4)	69.8 (72.3)	7145 ± 3370	5964 ± 3778	2.8 ± 1.2	2.7 ± 1.0	16.4 ± 1.6	16.6 ± 1.6	6.6 ± 0.7	6.6 ± 0.9
6	49	50	112.9 (86.3)	95.2 (53.9)	7533 ± 3735	7888 ± 3856	2.5 ± 0.9	2.9 ± 1.2	16.1 ± 2.2	16.9 ± 1.9	6.7 ± 0.6	6.9 ± 0.8
7	53	53	76.1 (58.4)	74.9 (62.2)	7905 ± 3763	7497 ± 3534	2.8 ± 1.5	2.3 ± 1.0	16.4 ± 1.6	16.9 ± 1.9	6.8 ± 0.7	6.8 ± 0.5
8	59	42	82.5 (52.7)	71.7 (51.1)	8523 ± 3840	7410 ± 3639	2.5 ± 0.9	2.7 ± 1.1	15.8 ± 1.5	16.6 ± 1.9	6.5 ± 0.7	6.8 ± 0.7
9	62	43	78.0 (70.8)	66.0 (50.8)	7507 ± 4069	8539 ± 4362	2.6 ± 1.1	2.6 ± 0.9	16.4 ± 2.0	16.1 ± 1.9	6.6 ± 0.9	6.8 ± 0.5
10	63	37	81.2 (52.7)	69.8 (59.6)	10,013 ± 4735	8135 ± 4872	2.8 ± 1.1	2.9 ± 1.0	15.9 ± 1.7	15.2 ± 2.0	6.6 ± 0.7	6.6 ± 0.6
11	70	59	81.2 (48.9)	76.1 (54.6)	10,593 ± 5385	10,108 ± 5001	2.7 ± 1.0	2.5 ± 1.1	15.4 ± 1.8	14.6 ± 1.7	6.6 ± 0.6	6.7 ± 0.6
12	59	58	73.6 (52.0)	83.1 (69.5)	10,969 ± 4639	11,704 ± 7545	2.5 ± 1.2	2.7 ± 1.4	15.0 ± 2.1	14.1 ± 1.7	6.7 ± 0.8	6.5 ± 0.7
13	81	65	72.3 (59.6)	80.0 (69.8)	12,138 ± 7323	13,093 ± 7053	2.8 ± 1.6	2.4 ± 1.1	14.8 ± 1.7	14.4 ± 1.9	6.9 ± 0.7	6.1 ± 0.8
14	74	46	82.5 (45.1)	85.7 (39.0)	15,401 ± 7242	16,118 ± 9349	2.6 ± 1.1	2.3 ± 0.9	15.3 ± 2.0	15.6 ± 2.1	6.7 ± 0.7	5.8 ± 0.6
15	52	38	82.5 (50.1)	85.7 (60.9)	18,886 ± 8609	15,089 ± 8033	2.4 ± 1.1	2.2 ± 1.0	16.2 ± 2.2	15.3 ± 2.4	6.5 ± 1.0	5.5 ± 0.7
16	28	41	99.0 (51.4)	72.3 (76.1)	19,339 ± 8884	14,030 ± 8437	2.5 ± 1.3	2.4 ± 1.0	15.8 ± 2.2	15.4 ± 1.9	6.4 ± 0.4	5.5 ± 0.6
17	26	37	66.6 (63.5)	72.3 (49.5)	15,692 ± 8254	13,388 ± 7579	2.4 ± 1.2	2.3 ± 1.2	16.8 ± 2.3	15.1 ± 2.1	6.1 ± 0.6	5.4 ± 0.6

Laboratory results of all participants by age and gender. Mean with standard deviation, median with interquartile range

*TSH* thyrotropin, *FT3* free triiodothyronine, *FT4* free thyroxine

**Table 2:** Distribution of spot urine iodine levels of all participants by age and gender according to the WHO classification

Iodine (μg/L)	< 20		20–49		50–99		100–199		200–299		≥ 300	
Age group (years)	Male	Female	Male	Female	Male	Female	Male	Female	Male	Female	Male	Female
*% of children in the corresponding age group*												
0	2.5	0.0	9.8	9.1	35.2	36.4	27.9	38.6	18.0	13.6	6.6	2.3
1	8.3	5.3	20.8	31.6	25.0	36.8	37.5	21.1	8.3	5.3	0.0	0.0
2	16.7	3.3	11.1	33.3	30.6	33.3	36.1	26.7	5.6	3.3	0.0	0.0
3	2.2	5.0	11.1	20.0	31.1	37.5	51.5	32.5	2.2	5.0	2.2	0.0
4	0.0	0.0	17.6	21.6	43.1	51.4	37.3	24.3	2.0	2.7	0.0	0.0
5	1.8	3.8	12.5	22.6	41.1	41.5	39.3	28.3	3.6	3.8	1.8	0.0
6	2.0	0.0	10.2	16.0	32.7	36.0	44.9	48.0	10.2	0.0	0.0	0.0
7	5.7	9.4	18.9	17.0	45.3	43.4	28.3	30.2	1.9	0.0	0.0	0.0
8	1.7	2.4	16.9	23.8	47.5	47.6	32.2	23.8	1.7	2.4	0.0	0.0
9	8.1	2.3	25.8	27.9	33.9	44.2	29.0	23.2	3.2	2.3	0.0	0.0
10	4.8	8.1	12.7	32.4	49.2	29.7	27.0	24.3	6.3	5.4	0.0	0.0
11	1.4	6.8	20.0	20.3	47.1	45.8	30.0	27.1	1.4	0.0	0.0	0.0
12	5.1	10.3	20.3	17.2	49.2	36.2	22.0	34.5	3.4	1.7	0.0	0.0
13	6.2	4.6	23.5	27.7	40.7	27.7	27.2	40.0	2.5	0.0	0.0	0.0
14	1.4	2.2	18.9	17.4	44.6	43.5	32.4	34.8	2.7	2.2	0.0	0.0
15	3.8	2.6	19.2	15.8	46.2	39.5	23.1	39.5	5.8	2.6	1.9	0.0
16	0.0	9.8	7.1	22.0	42.9	36.6	50.0	29.3	0.0	0.0	0.0	2.4
17	11.5	0.0	26.9	35.1	26.9	43.2	34.6	18.9	0.0	2.7	0.0	0.0
All age groups	4.1	4.4	16.7	22.1	40.6	39.4	32.3	31.2	5.2	2.7	1.1	0.3

Spot urine iodine distribution of all participants sorted by the WHO classified categories (< 20 µg/L severe iodine deficiency, 20–49 µg/L moderate iodine deficiency, 50–99 µg/L mild iodine deficiency, 100–199 µg/L adequate iodine nutrition, 200–299 µg/L above requirements, ≥ 300 µg/L excessive). Percentage refers to the number of children in the corresponding age group. Last line presents percentage of all children (every age) in the corresponding WHO category. In summary 61.2% of boys and 65.9% of girls presented with spot urine iodine concentration below 100 µg/L

### Creatinine adjusted iodine spot levels

**Fig. 2:**
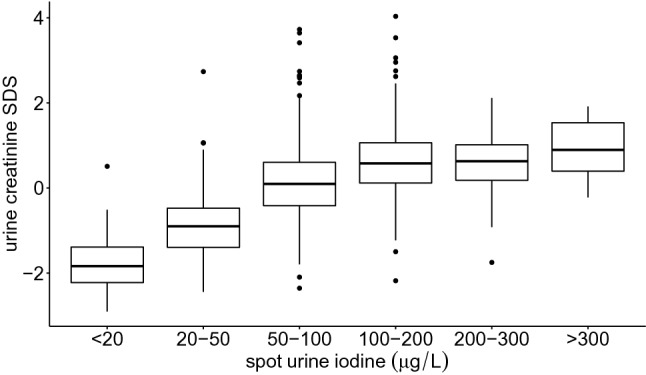
Creatinine SDS and spot urine iodine levels of all participants according to the WHO classification. Creatinine-SDS were calculated for each age group and compared to WHO iodine categories. Participants with higher urine creatinine levels also presented higher iodine levels

Low SUIC values were associated with low creatinine levels (Fig. 2). Therefore, age- and sex-specific CCUI values were calculated. The data indicated a significant correlation between the two parameters (iodine SDS alone and CCUI-SDS) although the correlation coefficient was low (*r* = 0.15, *p* < 0.001; Table 3).

**Table 3:** Pearson correlation coefficient and linear regression model calculation for age, iodine status and thyroid function of all participants

Compared data	Correlation coefficient		Linear regression	
	Corr	*p*	beta	*p*
Age vs. SUIC	− 0.19	< 0.001	− 0.02	< 0.001
Age vs. SUIC (only > 1 year)	− 0.08	0.33	− 0.006	0.006
Age vs. TSH	− 0.20	< 0.001	− 0.77	< 0.001
Age vs. fT3	− 0.32	< 0.001	− 1.82	< 0.001
Age vs. fT4	− 0.24	< 0.001	− 0.56	< 0.001
Age vs. creatinine (urine)	0.62	< 0.001	< 0.001	< 0.001
SUIC-SDS vs. TSH-SDS	− 0.04	0.11	− 0.04	0.11
SUIC-SDS vs. fT3-SDS	− 0.07	0.02	− 0.05	0.06
SUIC-SDS vs. fT4-SDS	− 0.01	0.63	− 0.01	0.74
SUIC-SDS vs. SUIC/creatinine-SDS	0.15	< 0.001	0.08	< 0.001
SUIC/creatinine-SDS vs. TSH-SDS	− 0.01	0.80	− 0.01	0.79
SUIC/creatinine-SDS vs. fT3-SDS	0.01	0.75	0.03	0.58
SUIC/creatinine-SDS vs. fT4-SDS	0.02	0.93	0.05	0.36

Calculated bivariate correlation coefficient (corr) with significance level (*p*) and linear regression model. SDS values were calculated age and gender dependent

*SUIC* spot urine iodine concentration, *TSH* thyrotropin, *FT3* free triiodothyronine, *FT4* free thyroxine

### Iodine deficiency and thyroid parameters TSH, FT3 and FT4

To investigate the impact of SUIC on thyroid function we analyzed serum thyroid biomarkers TSH, FT3 and FT4 of all participants (Table 1). All three biomarkers, as well as urine creatinine levels, presented a typical age-dependent distribution: TSH decreased during childhood with highest levels in the first year and lowest levels at the age of 17 years. FT3 and FT4 also decreased in early childhood, but thereafter, FT4 presented a small rise in older adolescents and FT3 showed a peak in boys at the age of 13 years. Urine creatinine increased significantly with increasing age. Pearson correlation coefficients were calculated between the SDS values for TSH, FT3, FT4 and those for SUIC and CCUI (Table 3). Only the association between FT3-SDS and SUIC was statistically significant.

**Fig. 3:**
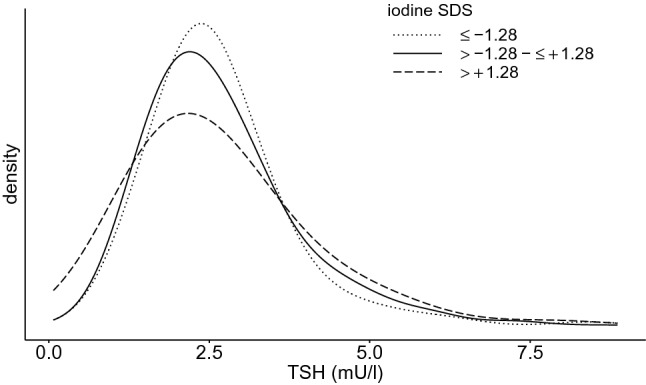
TSH distribution in high-, medium- and low- spot urine iodine groups. TSH (thyrotropin) distribution in three different groups [small dots: individuals with low spot urine iodine (< − 1.28 SDS), continuous line: individuals with medium spot urine iodine (− 1.28 to + 1.28 SDS), broad dots: individuals with high spot urine iodine (> + 1.28 SDS)]. TSH is slightly shifted towards right side in individuals with lower iodine excretion

**Fig. 4:**
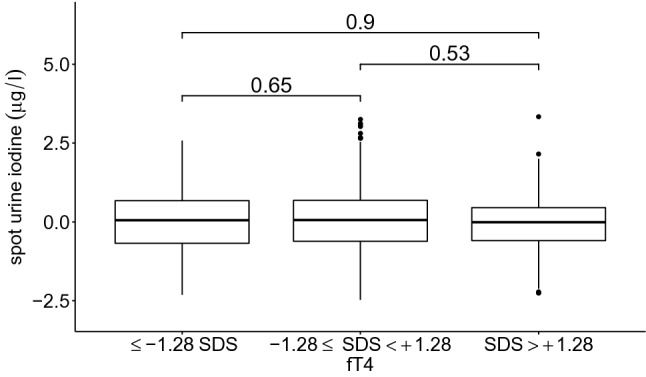
Spot urine iodine in high-, middle- and low-FT4 groups. Participants were separated into three groups based on FT4 (free thyroxine) level (< − 1.28 SDS, − 1.28 to + 1.28 SDS, >  + 1.28 SDS) and their spot urine levels compared. The numbers on the bars indicate the significance level. Individuals with different FT4 levels presented no significant difference in iodine excretion

Moreover, SUIC values with low (< − 1.28), moderate (− 1.28 to + 1.28) and high (>+ 1.28) SDS were compared in respect to levels of TSH (Fig. 3), FT3 and FT4 (Fig. 4) in the same participants. Again, in relation to thyroid biomarker levels, no significant difference was identified between subjects with low, moderate and high SUIC.

In our cohort, 3.7% of participants presented increased (*n* = 59, subclinical hypothyroid) or decreased TSH (*n* = 7, subclinical hyperthyroid) compared with reference values from an in-house study [23]. Of course, this number is very low but in the interests of control we compared these subjects with the whole cohort. SUIC-SDS values of − 0.02 (*p* = 0.85) and + 0.52 (*p* = 0.19) suggest that these subjects do not present any significant difference in this regard. Finally, in our study, 24 participants were excluded due to the identification of mild goiter during the medical examination. But even with these individuals, when the SUIC data are compared with those for the whole cohort, the values are close to the median level and largely comparable (+ 0.08 SUI-SDS).

### Iodine deficiency and thyroid autoantibodies

Levels of thyroid antibodies (TPO-Ab and TG-Ab) were measured in all subjects (data not shown). The antibody values demonstrated very little in the way of age-dependent variation during childhood and adolescence. Nor were there significant correlations for TPO-Ab or TG-Ab and SUIC. Furthermore, a comparison of the highest and the lowest iodine tertiles produced comparable SUIC levels (*p* > 0.05).

## Discussion

### Spot urine iodine levels

The first stated aim of our study was to investigate the distribution of SUIC values for a typical cohort of healthy, Central European children and adolescents without chronic or acute disease measured according to the WHO recommendations (Table 1; Fig. 1). Surprisingly, more than half the SUIC values for our cohort were below the WHO recommended median of > 100 µg/L, with values for 61.39% of the boys and 65.91% of the girls below this target. Additionally, the percentages of boys (20.79%) and girls (26.52%) with SUIC < 50 µg/L were higher than the 20% target figure indicated in the WHO recommendations. The reasons for such a conspicuous undershooting of the WHO targets remain to be debated. Despite many efforts to obtain an representative distribution of data our study population shows a bias towards a higher educational and socioeconomic status what may be an important limitation [19, 20]. Our samples were taken in the morning and in fasting state in accordance with WHO recommendations. Therefore, our data are comparable to other worldwide surveys about iodine distribution in urine [2, 7, 12]. In line with other surveys, our data summarize a negative trend in iodine supply for Central Europe in the last 20 years. In 1999, a nationwide survey in healthy German 6- to 12-year-old school children reported a suboptimal iodine intake in 27% [13]. Another nationwide study disclosed that 41% of children and adolescents revealed a suboptimal iodine intake between 2003 and 2006 [12]. In the same period, the DONALD study from the area of Dortmund in Western Germany described a significant decrease of iodine excretion by approximately 1 µg/day per year. A lower use of iodized salt in industrial food production was discussed as a potential reason for this observation [26, 27].

The highest SUIC values were observed in children in their first year of life. These high values of infants may be explained by higher iodine intake during pregnancy and breastfeeding. Both of these periods are characterized by an increased need for iodine, vitamins and other micronutrients and both the WHO and the American Academy of Pediatrics recommend supplementation of these substances [28–30]. An increased iodine intake may be attributable to mineral supplements, micronutrient-enriched breast milk and/or enriched formula. It is, therefore, likely that the high SUIC values in the first year of life are the result of increased intake. This hypothesis is supported by the observation that the majority of the subjects in our cohort with excessive values for SUIC were less than one year of age. During early childhood, SUIC levels were decreasing and thereafter remained at a relatively constant level with a small peak during puberty. Such a variance in SUIC could be probably caused by a change in dietary habits [31]. Previous studies described a significant gender difference in SUIC levels, with higher values in boys [32]. We could not confirm this finding in our cohort.

It is important to remember that iodine in urine has a short half-life after ingestion. About 90% of the iodine is eliminated within 24–48 h after intake [15]. Measurements of iodine excretion in patients with radioiodine therapy revealed that after admission, 66% of iodine could be detected in urine after 24 h, 87% after 48 h and 99% after 120 h [33]. Therefore, the individual iodine urine concentration only reflects iodine ingested in the most recent hours or days. To overcome this issue, some authors suggest measuring SUIC after multiple sampling or in 24 h-pooled samples [34, 35]. Furthermore, in terms of SUIC values, repeated 24 h-sampling over a number of months is more reproducible, whereas a repeated collection of spot urine produces greater variation [36]. The most important advantage of spot urine collection vs. 24 h urine sampling is its practicability. Even the WHO recommends spot urine measurements because only small amounts of urine are required for the SUIC analysis and casual urine specimens are easy to obtain [2]. In large studies, in particular, it is practically impossible to collect 24 h-pooled samples and, in any case, as the number of participants involved in the study increases, the day-to-day and the intra-day variation of SUI levels tends to even out [2]. Moreover, the classification of a cohort as iodine sufficient or insufficient by the measurement of SUIC has been shown to exhibit excellent reproducibility over one month [36, 37]. As such, SUIC measurement is not appropriate for indicating iodine deficiency in a single individual, but it is very useful for comparing different cohorts or monitoring long-term changes.

### Creatinine adjusted iodine spot levels

Published research indicates that, in children, CCUI matches better with 24 h-iodine excretion than uncorrected spot urine values spot urine alone [38]. As described above, we used age- and gender-dependent SDS values for CCUI as creatinine concentration in urine was found to increase noticeably with age. Additionally, the presence of a strong relationship between urine creatinine-SDS and SUIC-SDS implies that hydration status may be an important consideration in the interpretation of iodine data. We, therefore, tested for a possible association between thyroid hormone parameters and both SUIC-SDS and CCUI-SDS. However, it should be noted that the use of CCUI has two important limitations. First, creatinine excretion depends on the individual’s muscle mass and nutritional status. Compared to well-nourished children, children suffering from malnourishment tend to have lower serum creatinine levels [39]. In this respect, creatinine-correction does not appear to be helpful, especially when comparing cohorts across different regions of the world. Second, creatinine in children is strongly age-dependent, whereas, as we have seen, iodine levels in our study demonstrated only a marginal degree of age dependency. As such, age has a much higher influence on the SUIC/creatinine-ratio than the iodine levels themselves. In consequence, age-adjusted CCUI-SDS values would be required for the statistical investigation of iodine data in different cohorts. It is also, therefore, mandatory that there be a large number of participants in each age group. Creatinine adjustment, however, may introduce an additional bias into the data. It is, therefore, not recommended when measuring iodine or other trace elements in urine [40]. The WHO uses SUIC measurements only, and describes the iodine to creatinine ratio as “cumbersome, expensive and unnecessary” [2]. In our cohort, the correlation between CCUI-SDS and SUIC-SDS was relatively weak. The reason is that creatinine in urine has a high impact on SUIC. Our conclusion is, therefore, that these parameters measure two different traits. The calculation of CCUI can give additional information about a cohort but in our study neither SUIC nor CCUI were associated with the thyroid function.

### Iodine deficiency and thyroid parameters: TSH, FT3 and FT4, and thyroid autoantibodies

Because SUIC levels in our cohort were unexpectedly low and clearly below the WHO recommendation, the question arises whether or not the inferred iodine deficiency was associated with features of an altered thyroid function. Concentrations of thyroid parameters presented a typical age-dependent distribution consistent with findings from other studies and as discussed above [41]. It is important to underline that all the children and adolescents included in our cohort were clinically healthy. Surprisingly, we were able to show that, despite the low SUIC values, there was no indication of significant impairment of thyroid function biomarkers in our cohort. Even if we adjusted iodine levels for hydration status, there was no detectable correlation with thyroid hormone data. Furthermore, the children with increased or decreased TSH levels (3.7%) showed iodine concentrations similar to those for the whole cohort. In the literature, a clear inverse relationship has been described between median SUIC and the prevalence of goiter in children as identified using ultrasound [42]. Where comparisons have been carried out between cases with iodine uptake of < 50 µg/L, and cases of iodine uptake of > 100 µg/L, an increased prevalence of goiter has been observed in the < 50 µg/L group. However, the data relating to changes in thyroid hormone parameters in cases of altered SUIC are inconsistent. A positive association with TSH has been described for high SUIC values [43]. It has also been shown that a higher iodine supply in a cohort can raise the levels of TSH reference ranges [44]. However, other authors have reported finding no difference in thyroid hormone parameters when comparing groups of patients with low (< 50 µg/L) as compared to high SUIC (> 200 µg/L) values [45, 46].

The WHO determined the recommended daily intake of iodine using a calculation of intake in healthy individuals combined with a body of experimental data [47]. The relationship between the daily intake and SUI concentration was based mostly on the hypothesis that an increased supply via nutrition can be absorbed and eliminated within a short time. A steady-state condition had already been demonstrated experimentally [48, 49]. However, no exact SUIC level has previously been identified, on the basis of clinical studies, as a critical threshold for subsequent thyroid dysfunction. Given that our clinically healthy cohort had an insufficient iodine intake according to the WHO recommendation it would appear that these recommended levels are higher than those at which a clinical threshold of this sort would be set. As mentioned above, the WHO suggested the use of spot urine for the ascertainment of SUIC data. Due to the large day-to-day and within-subject variance of iodine levels, there is a possibility of overestimating the number of persons with deficiency or excessive intake. Other methods have been proposed for calculating the true prevalence of deficiency/excess, such as the EAR/UL method [50].

Additionally, we investigated the relationship between SUIC values and levels of thyroid autoantibodies. It has been suggested, somewhat controversially, that higher iodine intake can induce autoimmune thyroiditis [51–53]. In our cohort, autoantibody levels of subjects with relatively high SUIC values were comparable with those in subjects with low SUIC values.

In summary, we investigated spot urine iodine concentration in clinically healthy children and adolescents. Our results were interpreted with reference to WHO recommendations and revealed SUIC values clearly below the recommended levels. However, low SUIC values in our cohort were not associated with impaired thyroid function. Our results suggest that the parameters used to define iodine deficiency for clinical applications need to be revisited.
